# Spectroscopic Characterization of the Binding and Release of Hydrophilic, Hydrophobic and Amphiphilic Molecules from Ovalbumin Supramolecular Hydrogels

**DOI:** 10.3390/gels9010014

**Published:** 2022-12-26

**Authors:** Ana Vesković, Danica Bajuk-Bogdanović, Vladimir B. Arion, Ana Popović Bijelić

**Affiliations:** 1 Faculty of Physical Chemistry, University of Belgrade, Studentski trg 12-16, 11158 Belgrade, Serbia; 2 Institute of Inorganic Chemistry, University of Vienna, Währinger Strasse 42, A-1090 Vienna, Austria

**Keywords:** binding and release kinetics, EPR spectroscopy, ovalbumin, Raman spectroscopy, supramolecular hydrogels

## Abstract

Protein-based hydrogels have attracted growing attention for pharmaceutical and biomedical applications. Ovalbumin (OVA), the hen egg white albumin, possessing good foaming and gelling properties and being widely used in the food industry, has recently been indicated as a potential pharmaceutical vehicle. In this study, the binding and release properties of pure OVA hydrogels were investigated by electron paramagnetic resonance (EPR) spin labeling. The comparative analysis between OVA and serum albumin (SA) hydrogels revealed the same release kinetics of hydrophilic 3-carbamoyl-proxyl and 3-carboxy-proxyl, suggesting the diffusion-dominated release of small probes from both hydrogel types. The results obtained with the amphiphilic 16-doxylstearate (16-DS) indicate that OVA, unlike SAs, does not possess a specific fatty acid binding site. However, the OVA hydrogels were able to accommodate a two-fold excess of 16-DS, resulting from protein thermally induced conformational changes, as confirmed by Raman spectroscopy. Similarly, the hydrophobic modified paullone ligand **HL**, which was initially free in the OVA solution, was bound in the hydrogel. The hydrogels were found to retain a significant amount of 16-DS and **HL** after 7-day dialysis in physiological saline. The observed facilitated binding of amphiphilic/hydrophobic molecules in OVA hydrogels compared to the solution, and their sustained release, demonstrate the applicability of OVA hydrogels in pharmaceutics.

## 1. Introduction

Ovalbumin (OVA), the albumin from hen egg white, accounts for more than half of its total protein content. It appears in the egg yolk only in its dephosphorylated form, presumably serving as the nutrition source for the growing embryo [1]. OVA belongs to the serpin superfamily, despite lacking any protease inhibitory activity. Although it is the first protein isolated in pure form from egg white, its physiological function has not been fully resolved [1,2]. It is mainly proposed that OVA serves as a transport and storage protein for amino acids and metal ions [1], possessing a strong-affinity binding site for bivalent cations [3]. This globular glycoprotein with ~45 kDa molecular mass is comprised of 385 amino acids, including six cysteines with a single disulfide bond [1,2]. Among its amino acid residues, about one-half are hydrophobic, and one-third are acidic, accounting for an OVA isoelectric point of 4.5 [4]. It is an ellipsoidal shape molecule of approximate dimensions 70 Å × 45 Å × 50 Å, with almost all of the polypeptide chain involved in the defined secondary structure. Native OVA consists of a mixture of α-helix and β-sheet structures and an exposed helicoidal loop [1,5,6]. 

The α-to-β structural transformation leading to OVA aggregation can be induced by thermal denaturation of the native protein [6], which commonly occurs during food protein processing [7]. By heating, high-pressure treatment, and enzymatic reactions, OVA can be easily modified for specific purposes [8]. Due to its distinctive functional properties, including foaming, gelling, and emulsifying [7,8], along with a well-balanced amino acid composition [9], availability, and low cost [10], this protein is widely used in the food industry. A current trend in the field of functional foods and pharmaceuticals is its application as a vehicle for bioactive compounds in various formulations [11,12].

OVA has been widely used as a model protein antigen in vaccine development due to its well-known immunogenic efficacy [13]. More recently, it has been found that ovalbumin epitope SIINFEKL (S: Serine, I: Isoleucine, N: Asparagine, F: Phenylalanine, E: Glutamic acid, K: Lysine, L: Leucine), commonly used to test new vaccine adjuvants, forms a supramolecular hydrogel, suggesting this peptide propensity to be the origin of its immunoactive properties [14]. 

Otherwise, OVA self-assembling into nanoparticles/gels under heating treatment [15,16] is slowly being recognized for its growing potential in biomedical applications. Besides, it has been reported that OVA exhibits anticancer, antihypertensive, antimicrobial, and antioxidant activities, as well as an antimutagenic activity when denatured by heat [17]. It has been shown that OVA-based nanoparticles could serve as an efficient carrier of epigallocatechin-3-gallate (EGCG) for ulcerative colitis therapy [15]. Furthermore, OVA-coated Fe_3_O_4_ nanoparticles have proved to be a good reservoir of chlorogenic acid, promoting its anticancer efficacy in vitro [18].

With the growing interest in natural hydrogels for drug delivery, the development of OVA hydrogels [16] is also gaining attention. Numerous systems for controlled delivery have been established in past decades, addressing issues related to conventional drug administration, including low efficacy, side effects, and toxicity [19]. The advantage of hydrogels over other platforms lies in their high water content, making them resemble biological tissues, and accounting for their excellent biocompatibility [20]. In this context, naturally derived hydrogels are particularly attractive, as they are commonly obtained by self-assembly physical crosslinking methods, modifying the temperature of the hydrogel precursor solution [21]. Unlike chemically cross-linked by covalent bonds, physical hydrogels are based on supramolecular interactions, including hydrogen bonding, hydrophobic interactions, van der Waals forces, electrostatic interactions, π-π stacking, host-guest interactions and metal ligand coordination [22,23,24,25], avoiding potentially toxic initiators and cross-linkers usage. Furthermore, as a potential advantage, supramolecular hydrogels exhibit responsive behavior through different physical states to external stimuli, such as temperature, pH, ionic strength, mechanical stress, or light [26].

As promising materials regarding safe injection/implantation, biocompatibility, and biodegradability, many studies are focused on protein-based hydrogels [27]. While serum albumins (SAs) have already proved to be suitable for the sustained release of different drugs [28,29,30,31], similar investigations of OVA hydrogels are lacking. However, in a recent study, the controlled release of curcumin from heat-induced OVA hydrogels was monitored, showing that acylated OVA hydrogels can serve as a vehicle for controlled delivery [16]. Furthermore, ovalbumin–pullulan hydrogels have demonstrated good delivery performance of curcumin under gastrointestinal digestion [12]. Regarding OVA interaction with drugs, it has been found that tetracyclines bind to OVA with high affinity, also inducing changes in the native protein structure [32].

Quite recently, it has been suggested that polyphenol binding provides a new way to regulate OVA self-assembly and gelling behavior, in the study of the effects of EGCG, an antioxidative component of various food products, on OVA gelation under heating treatments [33]. Additionally, the ionic surfactant-mediated ordered protein condensation (PC) [34] has been demonstrated on water-soluble hen egg white (HEW) proteins. Subsequently, thermally treated PC(HEW) has been shown to form a hydrogel with high mechanical strength, attributed to synergistic effects of non-covalent and covalent networks constructed by disulfide bonds between denatured OVA proteins [35].

**Scheme 1:**
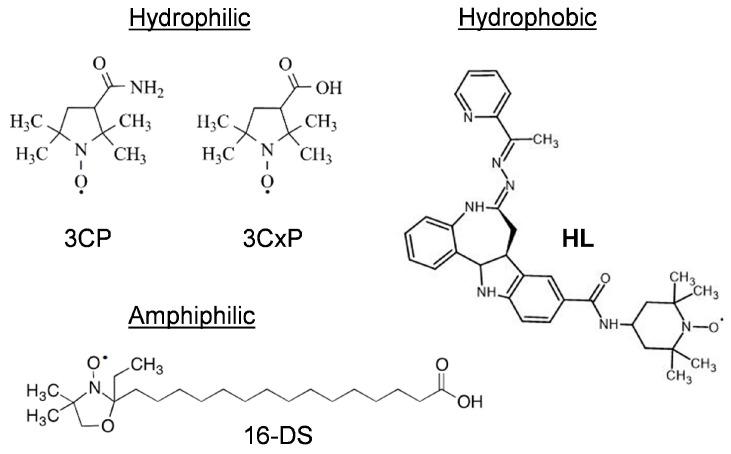
The structures of the hydrophilic, hydrophobic and amphiphilic spin-labeled substances used for OVA binding and release studies: 3-carbamoyl-2,2,5,5-tetramethylpyrrolidine-1-oxyl (3CP), 3-carboxy-2,2,5,5-tetramethylpyrrolidine-1-oxyl (3CxP), 16-doxyl-stearic acid (16-DS), and modified 7,12-dihydroindolo [3,2-d][1]benzazepin-6(5H)-one bearing the spin label 2,2,6,6-tetramethylpiperidine-1-oxyl (TEMPO) free-radical (**HL**).

This study was focused on pure thermally induced OVA hydrogels, ensuring their biocompatibility for safe, potential applications in pharmaceutics and functional food development. The capacity of supramolecular OVA hydrogels to bind and release selected model molecules was investigated by electron paramagnetic resonance (EPR) spin labeling, using four different spin-labeled compounds: the pyrrolidine nitroxides 3CP (hydrophilic, neutral) and 3CxP (hydrophilic, charged), the spin-labeled stearic acid 16-DS (amphiphilic), and the cytotoxic modified paullone ligand bearing a TEMPO free radical **HL** (hydrophobic) (Scheme 1). EPR spectroscopy is unsurpassed for ligand binding and release studies compared to other physicochemical techniques due to its nanomolar detection limit, and the requirement of extremely small sample volumes (30 µL), as well as the experiment time efficiency. However, its use is limited by the fact that it requires the covalent modification of the molecule with a stable radical (EPR-active group). Regarding the investigation of hydrogels, EPR spin labeling has been previously shown to be useful for controlled release studies of TEMPO-labeled coumarin-3-carboxylic acid and warfarin [36], and a spin-labeled naproxen derivative [37] from bovine serum albumin (BSA)-based hydrogels, as well as the cytotoxic ligand **HL** used as the model molecule for the release behavior of hydrophobic drugs from the human serum albumin (HSA) hydrogels [38]. Spin-labeled stearic acid (16-DS) has been utilized as a model molecule for the release behavior of amphiphilic drugs from BSA hydrogels [39], as well as for the confirmation of the existence of basic molten globule states of BSA hydrogels [40]. Finally, EPR spectroscopy has also been applied for the investigation of the suitability of aminoxyl spin probes to be used for hydrogel water content determination of BSA hydrogels [41] and for the study of the accessibility of a series of spin probes with molecular weights in the range of 200–60,000 Da in different polysaccharides and β-cyclodextrin-based hydrogels [42].

Moreover, the change in OVA secondary structure upon thermally induced gelation was investigated by Raman spectroscopy, which has been successfully employed for the study of the differences in structures between native OVA and its heat-stable form, S-OVA [43,44].

## 2. Results and Discussion

### 2.1. The Binding of Hydrophilic Spin Probes 3CP and 3CxP to OVA

**Figure 1:**
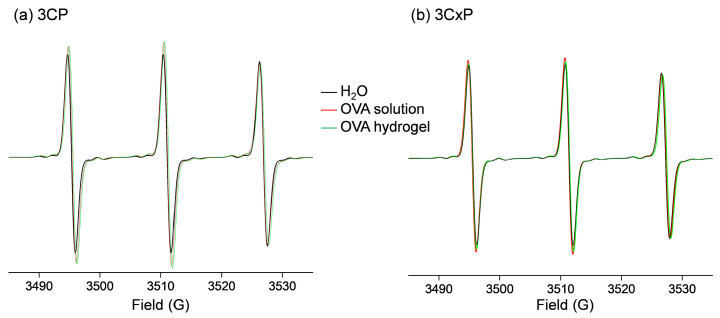
The X-band EPR spectra of (**a**) 3CP and (**b**) 3CxP in water (black), 30 wt% OVA solution (red), and the corresponding OVA hydrogel (green). The spectra were normalized with respect to the third line in the signals of 3CP and 3CxP, for comparison.

The EPR spectra of 3CP and 3CxP in water, 30 wt% OVA solution and the corresponding thermally induced hydrogel (Figure 1) display three-line EPR signals that arise from the aminoxyl group. The spectral parameters (line heights and widths) for each spin probe are slightly different in water and the protein environment as the result of their different rotational mobility. This may be quantified by the rotational correlation time (*τ_c_*), defined as the time it takes a free radical to rotate one radian around its axis [45], and calculated using Equation (1), given in Figure S1. The somewhat higher values of *τ_c_* (Table 1) obtained for 3CP and 3CxP in the OVA solution compared to water indicate a more restricted motion of the spin probes in the presence of the protein, as the result of increased viscosity of the solvent [46,47,48]. Furthermore, based on spectral analysis, it is found that 3CP and 3CxP do not bind to OVA, and in the hydrogel, they are located in the water pores. The same result has been obtained for the interaction of the neutral hydrophilic spin probe 3CP with BSA [41]. However, the charged spin probe 3CxP, which contains the carboxylate group, has been found to interact with BSA in solution via electrostatic forces [41]. This is also confirmed by a higher value of *τ_c_* previously obtained for 3CxP/BSA compared to 3CxP/OVA (Table 1), demonstrating stronger immobilization of this spin probe in presence of BSA. This result is most probably due to the lower isoelectric point of OVA [49] compared to BSA [50].

**Figure 2:**
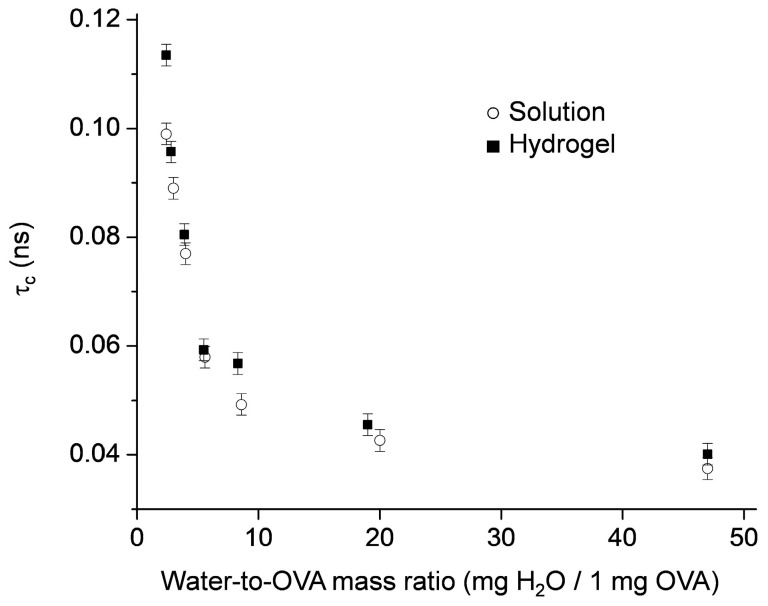
The calculated rotational correlation times for 0.5 mM 3CP in different wt% OVA solutions and corresponding hydrogels, plotted as the function of water-to-OVA mass ratio. Error bars are the standard deviation from two independent measurements.

To investigate further if the interaction of 3CP with OVA is similar to that with BSA, EPR spectra of 2, 5, 10, 15, 20, and 25 wt% OVA solutions and the corresponding hydrogels were measured. It should be highlighted here that the 2 wt% OVA solution was able to form a hydrogel, unlike the 2 wt% BSA solution [41]. The calculated rotational correlation times are shown in Figure 2 as a function of water-to-OVA mass ratio. The obtained correlation between *τ_c_* and the hydrogel water content was in full agreement with that found previously for the BSA hydrogels [41] (Figure S2), confirming that 3CP is located only in the water pores of the OVA hydrogel.

### 2.2. The Release of Hydrophilic Spin Probes 3CP and 3CxP from OVA Hydrogels

**Figure 3:**
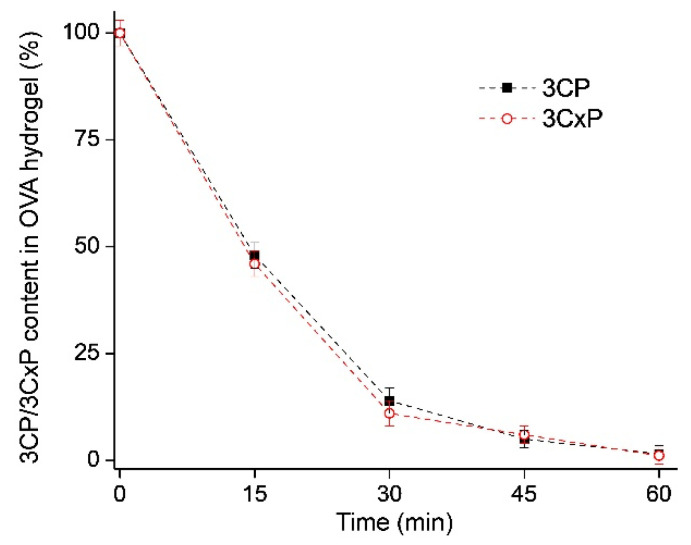
The rate of spontaneous release of 0.5 mM 3CP (black squares) and 0.5 mM 3CxP (red open circles) from 30 wt% OVA hydrogels in physiological saline at room temperature. Error bars are the standard deviation from two independent measurements.

The release of the hydrophilic spin probes that were shown to be entirely dissolved in the water phase of the hydrogel, not interacting with the protein matrix, was investigated in the 30 wt% OVA hydrogels. The 3CP/OVA and 3CxP/OVA hydrogels (~20 mg) were incubated (separately) in 50 mL of physiological saline at room temperature for 1 h. Every 15 min, the hydrogels were taken out of the physiological saline, carefully dried of excess saline, and placed in the EPR cell for whole-hydrogel EPR measurements. It was determined that the diffusion kinetics are the same for both spin probes, resulting in ~1% spin probe content in the OVA hydrogels after 1 h (Figure 3). This is in agreement with the result obtained for the release of 96% 3CP from the 30 wt% HSA hydrogel after 1 h [38], showing that the diffusion of hydrophilic molecules, which do not bind to the protein matrix, from OVA and HSA hydrogels, occurs on the same time scale. In this context, since it has been shown that the diffusion of the entrapped molecule throughout the hydrogel depends on the relative size of the molecule compared to that of the water pore [19,51,52], it may be concluded that the water pores of the supramolecular OVA and HSA hydrogels are larger than 3CP and 3CxP molecules, resulting in diffusion-dominated release of small probes from both hydrogel types [19]. It is worthy pointing out that the macroscopic appearance of the OVA hydrogels had changed upon incubation in physiological saline from transparent to opaque white (Figure S3), which has not been previously observed for HSA hydrogels [38]. Moreover, the study on PC(HEW) [35] has reported that upon immersion in water, the ionic surfactant-modified hen egg-white protein condensate hydrogels change their appearance from clear to opaque, which has been attributed to the regeneration of the non-covalent gel network.

### 2.3. The Binding of Amphiphilic and Hydrophobic Molecules to OVA

#### 2.3.1. Spin-Labeled Fatty Acid 16-DS

**Figure 4:**
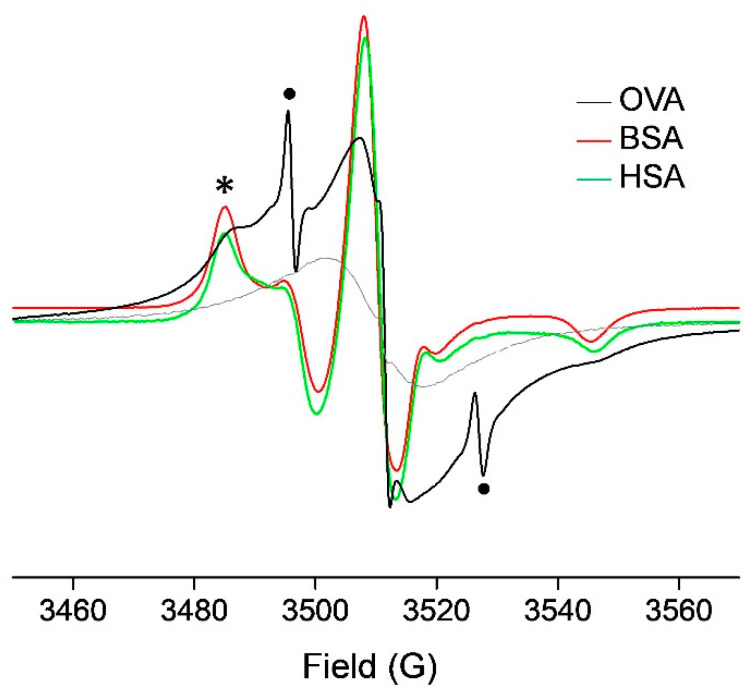
The X-band EPR spectra of 16-DS in solution with OVA (black), BSA (red) and HSA (green) for the molar ratio 16-DS:protein = 2:1. The signal contributions are marked as follows: 16-DS micelle in water (grey line), free 16-DS in water (•), and protein-bound 16-DS (∗).

The ability of OVA to bind fatty acids was investigated using 16-DS, a stearic acid spin-labeled at the C-16 atom of the hydrocarbon chain. The EPR spectrum of the OVA solution incubated with two equiv. of 16-DS (Figure 4, black line) displays large contributions that arise from the 16-DS micelle (Figure 4, grey line) and the free (protein-unbound) 16-DS (marked with •), and a small contribution from the protein-bound 16-DS (marked with ∗). The formation of the micelle could be expected, since the concentration of the fatty acid in the experiment was far above its critical micellar concentration [53]. On the other hand, when HSA and BSA were incubated with the same amount of 16-DS, the micelle and the free 16-DS were absent in the spectra (Figure 4, red and green lines) and only the contribution from the strongly immobilized spin label was observed, indicating strong binding of 16-DS to both proteins. In fact, it has been shown that BSA and HSA can bind at least six equiv. 16-DS [54,55]. This is in line with the fact that SAs serve as fatty acid transport proteins, and by binding to them, increase the solubility of fatty acids in blood plasma [29], suggesting that this is not the physiological function of OVA.

**Figure 5:**
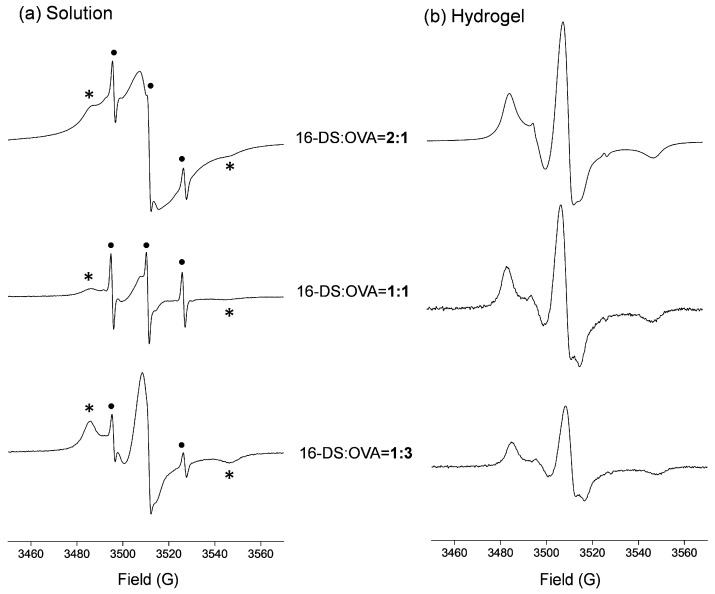
The X-band EPR spectra of 16-DS in OVA (**a**) solutions and (**b**) corresponding hydrogels, for different molar ratios 16-DS:protein, 2:1 (top), 1:1 (middle) and 1:3 (bottom). The signals that arise from the unbound 16-DS are marked with (•), and the signals that arise from the bound 16-DS are marked with (∗).

In further experiments, OVA was incubated with one equiv. 16-DS, with the aim to determine if the protein contains a specific binding site for the fatty acid. The resulting EPR spectrum showed some contribution from the protein-bound spin label, however most of 16-DS remained unbound (Figure 5a, middle spectrum). Finally, when OVA was incubated with 0.33 equiv. 16-DS, the EPR spectrum showed a marked relative increase in the signal that arises from the protein-bound spin label, although still exhibiting a contribution from the unbound label. This indicates that OVA does not possess a specific binding site for the fatty acid, like BSA and HSA [56], and that 16-DS non-specifically binds to OVA, either by electrostatic interactions via the carboxylic head, or by hydrophobic interactions via the hydrocarbon tail.

In contrast, the supramolecular OVA hydrogel was found to accommodate even two equiv. 16-DS. Namely, the EPR spectra of two, one, and 0.33 equiv. 16-DS in the OVA hydrogel exhibit only the contribution from the strongly bound spin label (Figure 5b). This is most probably the result of protein conformational changes that take place during OVA thermal denaturation, which results in the formation of the hydrogel with more exposed protein hydrophobic regions. A similar finding has been obtained for heat-induced nanometric OVA aggregates, which were shown to bind 1.4–2.0× more linoleic acid than native OVA [57].

#### 2.3.2. Spin-Labeled Cytotoxic Ligand **HL**

**Figure 6:**
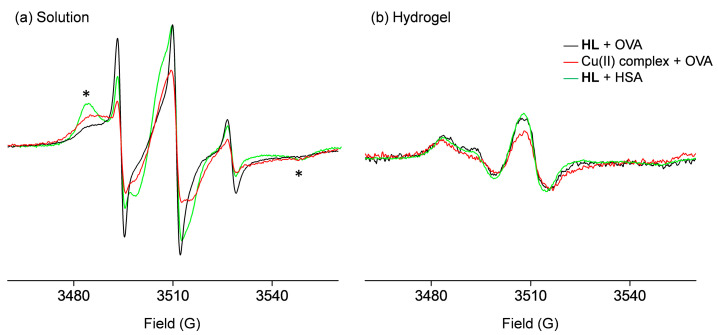
The X-band EPR spectra of 0.65 mM **HL**/30 wt% OVA (black), 0.65 mM copper(II) complex of **HL**/30 wt% OVA (red), and 0.5 mM **HL**/30 wt% HSA (green), in (**a**) solution and (**b**) corresponding hydrogel. The signals that arise from **HL** bound to the protein are marked with (∗).

The spin-labeled compound used to probe the OVA binding capacity for hydrophobic molecules was **HL**, a modified paullone ligand bearing a TEMPO free-radical unit, which exhibits extraordinary antiproliferative activity in human cancer cell lines [58]. The EPR spectrum of **HL** incubated with a solution of OVA for 30 min at room temperature indicates that **HL** binds to the protein only to a small extent, and that most of **HL** remains unbound. Compared to HSA, it was observed that **HL** binds less strongly to OVA (Figure 6a, compare black and green lines) for the same molar ratio **HL**:protein =1:10. Recently, it has been suggested that **HL** binds to HSA via the paullone backbone, within the drug-binding Sudlow site 2, with moderate affinity (formation constant logK ~ 3.4 ± 0.2) [38]. It is, therefore, very likely that OVA does not possess a specific binding site for **HL**. Furthermore, the binding of the copper(II) complex of **HL** to OVA was found to be stronger than the ligand alone (Figure 5a, red line), suggesting that the Cu^2+^ ion may play a role in its binding. It has been shown that Cu^2+^ binds to OVA [59], quite possibly to the metal binding site, which was identified by ^31^P NMR measurements as high-affinity, for both Mn^2+^ and Zn^2+^ [3].

Finally, as observed for 16-DS, the OVA hydrogel was able to accommodate **HL** that was initially unbound in the solution. The binding of **HL** and its copper(II) complex in the OVA hydrogel appeared to be the same as the binding of **HL** to HSA (Figure 6b). It has been previously shown that **HL** binds more strongly in the HSA hydrogel compared to the solution as the result of the protein conformational change during the heating process, in which, the protein becomes more accessible to the ligand [38,60,61]. Therefore, it is probable that the conformation of OVA upon thermal denaturation, which contains more exposed hydrophobic regions [57,62,63], is better suitable for the accommodation of the ligand. This property of OVA supramolecular hydrogels is important for their potential applications as drug carriers, as well as for the production of functional foods, in which, it would significantly increase the aqueous solubility, and as shown for curcumin, the photostability, of hydrophobic active substances [11].

### 2.4. The Release of Amphiphilic and Hydrophobic Molecules from OVA Hydrogels

#### 2.4.1. Spin-Labeled Fatty Acid 16-DS

**Figure 7:**
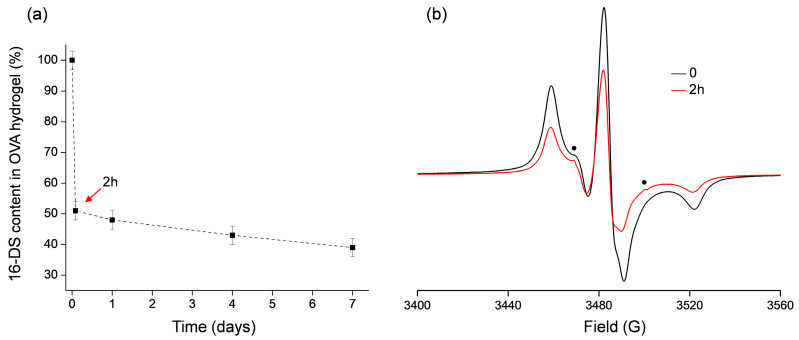
(**a**) The rate of spontaneous release of 16-DS from the OVA hydrogels in physiological saline at room temperature. Error bars are the standard deviation from two independent measurements. (**b**) The X-band EPR spectra of 16-DS/OVA hydrogels before (black) and after 2 h incubation in physiological saline (red). The small contribution, in the red spectrum, that arises from the free 16-DS is marked with (•).

The release of 16-DS from the OVA hydrogel was investigated using the same protocol used for the hydrophilic spin probes. Specifically, the 16-DS/OVA hydrogel (~20 mg) was incubated in 50 mL of physiological saline at room temperature for 7 days. At selected time points, the hydrogel was taken out of the physiological saline, carefully dried of excess saline, and placed in the EPR cell for whole-hydrogel EPR measurements. It was determined that after the first 2 h of incubation in saline, ~50% 16-DS was spontaneously released from the hydrogel (Figure 7a). After that, the rate of release was significantly reduced, resulting in only ~10% additional 16-DS release after 7 days.

The fast initial release of 16-DS on the time scale of hours, followed by a slow release over the course of a week, indicates that 16-DS was entrapped in the OVA hydrogel in at least two different-affinity sites. In line with this, the EPR spectrum of the OVA hydrogel measured after 2 h exhibits a very small contribution from the free 16-DS (Figure 7b, red line, marked with •), which was not observed in the spectrum of the as-prepared hydrogel (Figure 7b, black line), providing some experimental evidence for the different binding sites of 16-DS in the OVA hydrogel at these two time points. Moreover, the EPR spectra of the hydrogel measured after 1, 4, and 7 days (data not shown) were identical to the spectrum measured after 2 h (only lower in intensity), revealing that the binding mode of 16-DS remained unchanged during this time period.

These findings are different from those previously observed for the HSA hydrogel, using another spin-labeled stearic acid (5-DS), for which no release was found during 7 days of storage in physiological saline. The markedly different release kinetics of the spin-labeled fatty acids from the OVA and HSA hydrogels are in line with the fact that HSA contains at least 7 high-affinity fatty acid binding sites [54], which again indicates that the physiological function of OVA is not the binding of fatty acids. However, the results of this study show that OVA hydrogels are able to retain a considerable amount of an amphiphilic molecule that does not specifically bind to the protein in solution for a significant time period and, therefore, may be potentially used as carriers for amphiphilic active substances. By increasing their solubility, OVA hydrogels definitively may improve the bioavailability of these substances, which has been demonstrated in vivo for OVA nanoparticles containing polyphenols for the treatment of ulcerative colitis [15].

#### 2.4.2. Spin-Labeled Cytotoxic Ligand **HL**

The amount of **HL** released from the 30 wt% OVA hydrogel was ~30% after one day of incubation in physiological saline, followed by additional ~7–8%, after four, and again after seven days (total release of ~45% **HL** after seven days). It is important to note that the described release behavior was determined for the hydrogel with the **HL**:protein = 1:10 molar ratio; therefore, the release kinetics cannot be compared with that of 16-DS. However, it may be compared with the previously reported displacement of **HL** from the HSA hydrogel with the same **HL**:protein molar ratio [38]. The HSA hydrogel was able to retain a higher percent of **HL** after 7 days (70%), most probably as the result of the binding of the ligand to the Sudlow site 2. Further experiments need to be carried out on OVA hydrogels with different **HL**:protein molar ratios to better understand the binding mode of this hydrophobic ligand. Additionally, special attention should be given to the binding of metal complexes of **HL**, since OVA possesses a specific metal binding site [57]. In any case, the results from this study indicate that OVA hydrogels may be suitable vehicles for the transport and sustained release of hydrophobic drugs.

### 2.5. Raman Spectroscopy of OVA Solution and Hydrogel

**Figure 8:**
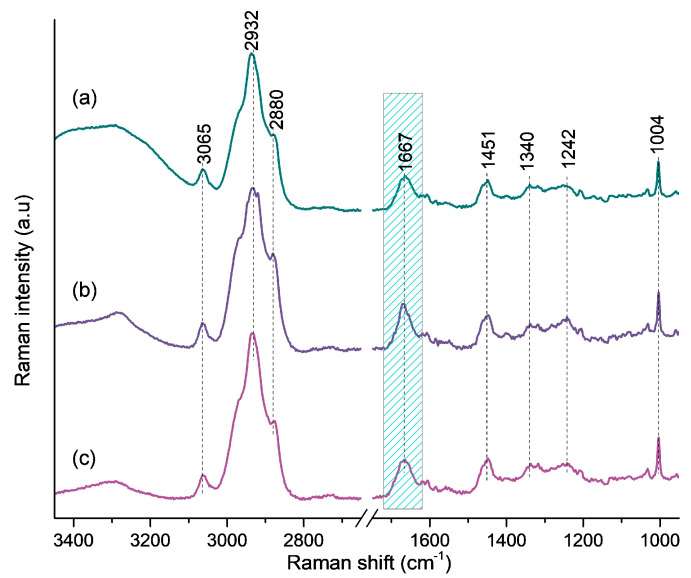
Raman spectra of OVA: (**a**) 30 wt% aqueous solution, (**b**) 30 wt% hydrogel, and (**c**) solid form.

**Figure 9:**
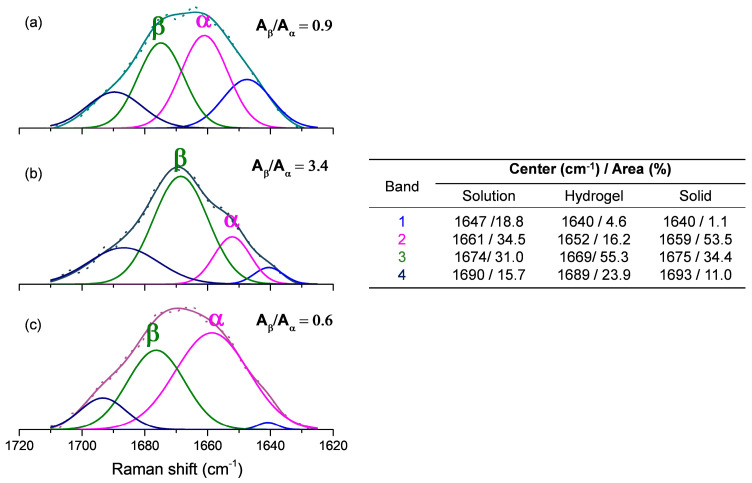
The deconvolution of the amide I band (1720-1620 cm^−1^ region) into individual subcomponents involving Gaussian curve fitting: (**a**) aqueous solution, (**b**) hydrogel, and (**c**) solid form. Inset table: Each band was assigned a maximum, and a percentage share of its surface area.

The observed facilitated binding of amphiphilic and hydrophobic molecules (16-DS and **HL**) in OVA hydrogels compared to OVA solutions was attributed to the existence of more exposed protein hydrophobic regions that are the result of conformational changes induced by the thermally induced gelation. To investigate the secondary structure changes in OVA upon thermal denaturation, Raman spectra of OVA in solution and hydrogel, as well as the solid (powder) form, were measured (Figure 8). The spectra of all samples showed bands in the C-H stretching region (3100–2800 cm^−1^). A band around 3065 cm^−1^ was assigned to aromatic residues, whereas bands at 2932 and 2880 cm^−1^ corresponded primarily to aliphatic amino acids [64]. The three vibrational modes are of main interest for the identification of different protein backbone conformations: amide I (stretching vibration of C=O), amide II, and amide III (both associated with coupled C–N stretching and N–H bending vibrations). The typical wavenumbers for amide I, II and III are 1690–1600 cm^−1^, 1580–1480 cm^−1^, and 1300–1230 cm^−1^, respectively [65]. In all samples, these bands are clearly visible, which indicates the preservation of the protein structure in different OVA forms. Upon closer inspection, it can be seen that the amide I band displays a slightly different shape in the three spectra (Figure 8, region marked in blue), and also that the band at 1242 cm^−1^ has the strongest intensity in the spectrum of the hydrogel. According to literature data, the Raman bands of α-helix and β-sheet structures are positioned at 1662–1655, and 1272–1264 cm^−1^ (α), 1674–1672, and 1242–1227 cm^−1^ (β), respectively, for amide I and amide III modes [65]. The analysis of individual bands obtained by deconvolution of the complex band in the amide I region gives insight into the presence of α-helix and β-sheet structures (Figure 9). As can be seen from the deconvolution parameters, the areas ratio A_β_/A_α_, of bands that are attributed to α-helix and β-sheet structures, is significantly higher in the OVA hydrogel (3.4), than in the other two OVA forms (solution 0.9, solid 0.6). This is consistent with the increase of intensity of the band at 1242 cm^−1^ in the amide III region, characteristic of β-sheets structure. Therefore, it can be concluded that the proportion of β-sheets is significantly higher in the OVA hydrogel compared to the OVA solution, as reported previously using Fourier transform infrared (FTIR) spectroscopy for the denatured form of OVA [66]. These results suggest that the facilitated binding of amphiphilic and hydrophobic molecules in OVA hydrogels are the result of the specific structure of the protein matrix. It is interesting to note that it has also been shown that the heat-stable form of OVA (S-OVA), which spontaneously and irreversibly forms during egg storage, also contains a higher contribution of the β-sheet structure compared to OVA [43,44]. It should be noted that the presence of 16-DS or **HL** in OVA hydrogels did not affect the aforementioned changes in secondary structure (Figure S4).

Additionally, the same samples were investigated by Attenuated total reflectance FTIR (ATR-FTIR) [67,68,69]. The ATR-FTIR spectra of OVA in solution and hydrogel, as well as the solid form display, like the Raman spectra, the characteristic peptide group bands: amide I (1634 cm^−1^), II (1533 cm^−1^), and III (1400–1200 cm^−1^) (Figure S5). However, the presence of water interferes with the protein spectrum in the amide I band region (the bending vibrational mode of water is ~1645 cm^−1^), which indicates that Raman spectroscopy is much more suitable for conclusive analysis of OVA secondary structure in solution and the hydrogel.

## 3. Conclusions

The binding of hydrophilic, hydrophobic, and amphiphilic compounds to OVA in solution and hydrogel, and their spontaneous release in physiological saline, were investigated by EPR spin labeling. The results show that the hydrophilic spin probes, 3CP (neutral) and 3CxP (charged), do not bind to OVA in solution, and in the hydrogel, they are located in the water pores. Upon storage in physiological saline, both spin probes diffuse out of the hydrogel within 1 h. The same diffusion kinetics of 3CP were reported previously for the HSA hydrogel, suggesting the comparable porosity of OVA and HSA hydrogels, which is of great importance for the design of OVA-based hydrogels intended for controlled drug delivery. 

The capacity of OVA to bind fatty acids was investigated using the spin-labeled stearic acid, 16-DS. It was shown that OVA does not possess a specific binding site for the fatty acid, and that the partial immobilization of 16-DS in the presence of OVA is most likely caused by incidental electrostatic interactions via the carboxylic head or by hydrophobic interactions via the hydrocarbon tail. This implies that the binding or transport of fatty acids, inherent for HSA and BSA, is not the primary physiological function of OVA. Nevertheless, the OVA hydrogels were able to accommodate a two-fold excess of 16-DS, most probably as the result of the protein conformational changes that accompany OVA thermal denaturation. Raman spectroscopy has confirmed that the secondary structures of OVA in solution and the thermally induced hydrogel are different, and that the proportion of β-sheets was significantly higher in the hydrogel.

The spin-labeled compound used to probe the OVA binding capacity for hydrophobic molecules was a modified paullone ligand bearing a TEMPO free-radical unit, **HL**. As observed for 16-DS, OVA does not seem to have a specific binding site for **HL** or for its copper(II) complex, although the binding of the complex was stronger, suggesting that the Cu^2+^ ion plays a role in its binding. Again, this indicates a marked difference between OVA and HSA, since, for the latter, it has been shown that **HL** binds within the drug-binding Sudlow site 2. Nevertheless, as shown for 16-DS, the OVA hydrogel was able to bind **HL** that was initially free in the solution. As already noted, the ability of OVA hydrogels to accommodate amphiphilic and hydrophobic molecules that do not bind to OVA in solution is most likely the result of the presence of exposed protein hydrophobic regions in its thermally denatured form. Finally, the OVA hydrogel was shown to retain a significant amount of 16-DS after 7-day dialysis in physiological saline, suggesting that OVA hydrogels are suitable vehicles for the transport and sustained release of amphiphilic molecules. This property may qualify OVA hydrogels to be appropriate for the same biomedical applications as SA hydrogels. Furthermore, the results regarding **HL** release from OVA hydrogels indicate that they may serve as hydrophobic drug carriers and also highlight their beneficial use in functional food production, in which their role would be to increase the aqueous solubility of hydrophobic active substances.

## 4. Materials and Methods

### 4.1. Materials

The thermally induced hydrogels were prepared from OVA (≥98%, Sigma-Aldrich, St. Louis, MO, USA), BSA (≥98%, Sigma-Aldrich, St. Louis, MO, USA), and HSA (≥99%, Sigma-Aldrich, St. Louis, MO, USA), in deionized water (Milli-Q, 18 MΩ·cm). The spin probes 3-carbamoyl-2,2,5,5-tetramethylpyrrolidine-1-oxyl free radical (3CP) (Sigma-Aldrich, St. Louis, MO, USA), 3-carboxy-2,2,5,5-tetramethylpyrrolidine-1-oxyl free radical (3CxP), (Sigma-Aldrich, St. Louis, MO, USA), 16-doxyl-stearic acid (16-DS) (Sigma-Aldrich, St. Louis, MO, USA), the spin-labeled modified paullone ligand (**HL**), and its copper(II) complex, were used for the investigation of OVA binding capacity. **HL** and the copper(II) complex of **HL** were synthesized as described in [58]. The synthesis and structure characterization details for both compounds are given in [58]. Physiological saline, 0.9% (*w/v*) NaCl solution (Hemofarm, Vršac, Serbia), was used as the incubation medium for the spin probe release studies.

### 4.2. EPR Spectroscopy

The binding of spin-labeled compounds to OVA in solution and corresponding thermally induced hydrogels, and their subsequent release during incubation in physiological saline, were measured by X-band EPR spectroscopy. The EPR spectra were acquired on a Bruker Biospin Elexsys II E540 EPR spectrometer with the following experimental parameters: microwave frequency 9.8 GHz, microwave power 10 mW, modulation amplitude 0.5 G, modulation frequency 100 kHz. For the binding studies, the samples for EPR measurements were drawn into 1 mm-diameter gas-permeable Teflon tubes (Zeus Industries Inc), and inserted into a quartz EPR cuvette (inner diameter 3 mm, Wilmad-LabGlass, Vineland, NJ, USA). For the release studies, the “macro” hydrogels (~20 mg) were placed in the ERP Tissue Sample Cell (Wilmad-LabGlass, Vineland, NJ, USA) for spectra acquisition.

### 4.3. Sample Preparation for the EPR Binding and Release Studies

The volume of all samples for the binding studies was 30 μL. For 3CP and 3CxP experiments, the samples contained 0.5 mM spin probe and 2, 5, 10, 15, 20, 25, or 30 wt% OVA solution. For 16-DS experiments, the samples contained 0.33, 1, or 2 mM 16-DS, and 1 mM OVA (or HSA, BSA). For the measurements of **HL** and the copper(II) complex binding, the samples contained 0.65 mM **HL** or its copper(II) complex and 30 wt% OVA, and 0.5 mM **HL** and 30 wt% HSA (note that the molar concentrations of 30 wt% OVA and 30 wt% HSA are different, due to their different molecular weights). The corresponding OVA hydrogels were prepared by incubation of the samples for 1 h at 80 °C [70]. BSA/HSA hydrogels were prepared from protein precursor solutions incubated at 75 °C for 40 min [41]. The homogenous distribution of the spin probes in the hydrogels was confirmed by EPR imaging, as described previously [38]. The release studies were performed on “macro” hydrogels (~20 mg) containing the same concentrations of spin probes and proteins as described for the binding studies. The hydrogels were incubated in 50 mL of physiological saline for selected time periods, after which, the hydrogels were taken out, carefully dried of excess saline, and placed in the EPR tissue cell for whole-hydrogel EPR measurements. The percentage of released spin probe from the hydrogel was determined by spin quantification (double integration) of the EPR signal [38,41].

### 4.4. Raman Spectroscopy

The Raman spectra were recorded on a Thermo DXR Raman microscope using the 532 nm laser excitation and constant laser power of 8 mW, grating with 900 lines/mm, and a spectrograph aperture of 50 μm pinhole. All samples were recorded under the same experimental conditions. The 30 wt% OVA solution (5 µL), and corresponding hydrogel (~10 mg) were placed on golden plates (Gold EZ spot micro, Thermo Fisher Scientific, Waltham, MA, USA), while the solid sample (~5 mg) was measured on a glass microscope slide. After acquisition, fluorescence background was subtracted from the Raman spectra using fifth order polynomial fit (OMNIC for Dispersive Raman 9.2.41 software). The selected spectral region was deconvoluted by applying the PeakFit™ version 4.12 software (SPSS Inc).

## Figures and Tables

**Table 1:** The EPR spectral parameters for 0.5 mM 3CP and 0.5 mM 3CxP in water, and 30 wt% OVA solution. The hyperfine splitting constants (*a*) expressed in Gauss, were determined from the spectra, and the rotational correlation times (*τ_c_*), expressed in nanoseconds, were calculated using Equation (1) in Figure S1.

Spin Probe	*a_1_* (G) ± 0.05	*a_2_* (G) ± 0.05	*τ_c_* (ns) ± 0.001
0.5 mM 3CP in H_2_O	16.00	16.00	0.031
0.5 mM 3CP in 30 wt% OVA	16.00	16.00	0.098
0.5 mM 3CP in 30 wt% BSA ^a^	16.02	16.03	0.113
0.5 mM 3CxP in H_2_O	16.10	16.20	0.036
0.5 mM 3CxP in 30 wt% OVA	16.24	16.30	0.085
0.5 mM 3CxP in 30 wt% BSA ^a^	16.25	16.27	0.137

**^a^** From reference [41].

## Data Availability

All data is contained in this article and its supporting information.

## Supplementary Materials

The following supporting information can be downloaded at: https://www.mdpi.com/article/10.3390/gels9010014/s1, Figure S1. The equation for rotational correlation time calculation (left) and the EPR spectrum of 0.5 mM 3CP in water (right), showing the following spectral parameters: w_0_—the linewidth of the central EPR line, and h_0_ and h_-1_—the heights of the central and high-field lines, respectively [45]; Figure S2. The calculated rotational correlation times plotted as the function of water-to-protein mass ratio, for 0.5 mM 3CP in different wt% OVA solutions and corresponding hydrogels (black), and compared to those obtained previously [41] for BSA (red); Figure S3. The macroscopic appearance of the 30 wt% OVA hydrogels (a) before and (b) after incubation in 50 mL physiological saline for 30 min at room temperature. Figure S4. Raman spectra of A: (a) 16-DS, (b) 30 wt% OVA aqueous solution, (c) 30 wt% OVA aqueous solution containing 16-DS in the molar ratio 1:1, (d) 30 wt% OVA hydrogel, and (e) 30 wt% OVA hydrogel containing 16-DS in the molar ratio 1:1; B: (a) HL, (b) 30 wt% OVA aqueous solution, (c) 30 wt% OVA aqueous solution containing HL in the molar ratio 10:1, (d) 30 wt% OVA hydrogel, and (e) 30 wt% OVA hydrogel containing HL in the molar ratio 10:1. Figure S5. ATR-FTIR spectra of OVA: (a) 30 wt% aqueous solution, (b) 30 wt% hydrogel, and (c) solid form.
