# Supplementary material for: Spectroscopic Characterization of the Binding and Release of Hydrophilic, Hydrophobic and Amphiphilic Molecules from Ovalbumin Supramolecular Hydrogels
Source: Gels. 2022 Dec 26;9(1):14. doi: 10.3390/gels9010014 (PMC9857392; doi:10.3390/gels9010014)
Supplement: Supplementary file 1 [file gels-09-00014-s001.zip › gels-2100028-supplementary.pdf]

Supplementary Material

for

Spectroscopic Characterization of the Binding and Release of Hydrophilic, Hydrophobic and Amphiphilic Molecules from Ovalbumin Supramolecular Hydrogels

Ana Vesković [1], Danica Bajuk-Bogdanović [1], Vladimir B. Arion [2], Ana Popović Bijelić [1,*]

[1] University of Belgrade – Faculty of Physical Chemistry, Studentski trg 12-16, 11158 Belgrade, Serbia

[2] Institute of Inorganic Chemistry, University of Vienna, Währinger Strasse 42, A-1090 Vienna, Austria

* Correspondence: ana@ffh.bg.ac.rs; Tel./Fax: +381-(0)-112187133

List of Figures:

Figure S1. The equation for rotational correlation time calculation (left) and the EPR spectrum of 0.5 mM 3CP in water (right), showing the following spectral parameters: w_0 – the linewidth of the central EPR line, and h_0 and h_{-1} – the heights of the central and high-field lines, respectively.

Figure S2. The calculated rotational correlation times plotted as the function of water-to-protein mass ratio, for 0.5 mM 3CP in different *wt*% OVA solutions and corresponding hydrogels (black), and compared to those obtained previously for BSA (red).

Figure S3. The macroscopic appearance of the 30 *wt*% OVA hydrogels (a) before and (b) after incubation in 50 mL physiological saline for 30 min at room temperature.

Figure S4. Raman spectra of **A**: (a) 16-DS, (b) 30 *wt*% OVA aqueous solution, (c) 30 *wt*% OVA aqueous solution containing 16-DS in the molar ratio 1:1, (d) 30 *wt*% OVA hydrogel, and (e) 30 *wt*% OVA hydrogel containing 16-DS in the molar ratio 1:1; **B**: (a) **HL**, (b) 30 *wt*% OVA aqueous solution, (c) 30 *wt*% OVA aqueous solution containing **HL** in the molar ratio 10:1, (d) 30 *wt*% OVA hydrogel, and (e) 30 *wt*% OVA hydrogel containing **HL** in the molar ratio 10:1.

Figure S5. ATR-FTIR spectra of OVA: (a) 30 *wt*% aqueous solution, (b) 30 *wt*% hydrogel, and (**c**) solid form.

1. Spin probe rotational correlation time

The spin probe rotational correlation time (τ_c), expressed in nanoseconds, can be calculated using Equation 1 in Figure S1 [1], from the following EPR spectral parameters: w_0 – linewidth of the central EPR line, expressed in Gauss, h_0 and h_{-1} – heights of the central and high-field lines, respectively, expressed in arbitrary units.

Equation 1.

$$\tau_c = 0.65 \cdot w_0 \cdot \left(\sqrt{\frac{h_0}{h_{-1}}} - 1\right)$$

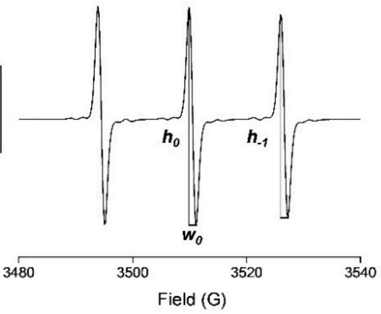

Figure S1. The equation for rotational correlation time calculation (left) and the EPR spectrum of 0.5 mM 3CP in water (right), showing the following spectral parameters: w_0 – the linewidth of the central EPR line, and h_0 and h_{-1} – the heights of the central and high-field lines, respectively.

2. The correlation between τ_c and the hydrogel water content of OVA and BSA hydrogels

The comparison of the calculated rotational correlation times as a function of water-to-protein mass ratio for OVA and BSA solutions and hydrogels is presented in Figure S2.

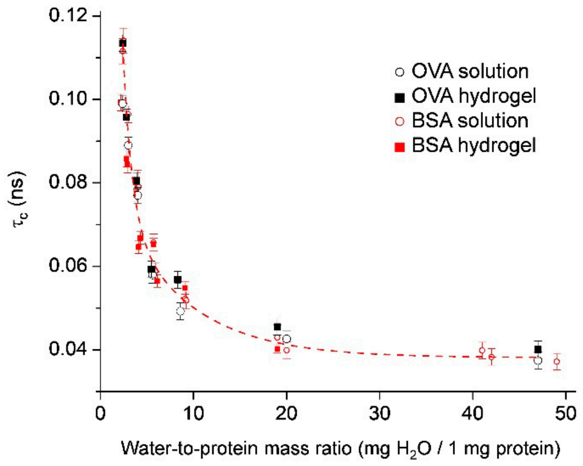

Figure S2. The calculated rotational correlation times plotted as the function of water-to-protein mass ratio, for 0.5 mM 3CP in different *wt*% OVA solutions and corresponding hydrogels (black), and compared to those obtained previously for BSA (red). Error bars are the standard deviation from two independent measurements. The data points for BSA fitted with a second order exponential fit ($y = 0.038 + 0.5 \cdot e^{-x} + 0.05 \cdot e^{-0.13x}$) were taken from [2].

3. Macroscopic appearance of OVA hydrogels

The macroscopic appearance of 30 *wt*% OVA hydrogels was changed upon incubation in physiological saline, from transparent to opaque white, as shown in Figure S3.

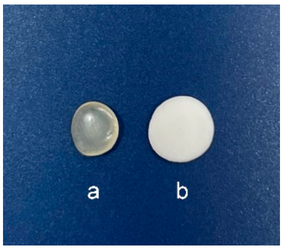

Figure S3. The macroscopic appearance of the 30 *wt*% OVA hydrogels (**a**) before and (**b**) after incubation in 50 mL physiological saline for 30 min at room temperature.

4. Raman spectra of OVA with 16-DS and HL

Raman spectra of the 30 *wt*% OVA solution and the corresponding hydrogel, containing 16-doxylstearic acid (16-DS) or the cytotoxic modified paullone ligand bearing a TEMPO free radical (**HL**), were measured with the aim to determine if the presence of the amphiphilic/hydrophobic molecule affects the changes in the OVA secondary structure observed upon gelation of pure OVA. As observed from the spectra shown in Figure S4, the amide I, II and III bands (1690–1600 cm^{-1}, 1580–1480 cm^{-1} and 1300–1230 cm^{-1}, respectively) do not exhibit changes in presence of 16-DS or **HL**. Also, the stronger intensity of the band at 1242 cm^{-1} observed in the spectrum of the pure OVA hydrogel compared to that of the OVA solution was maintained in the presence of investigated spin probes.

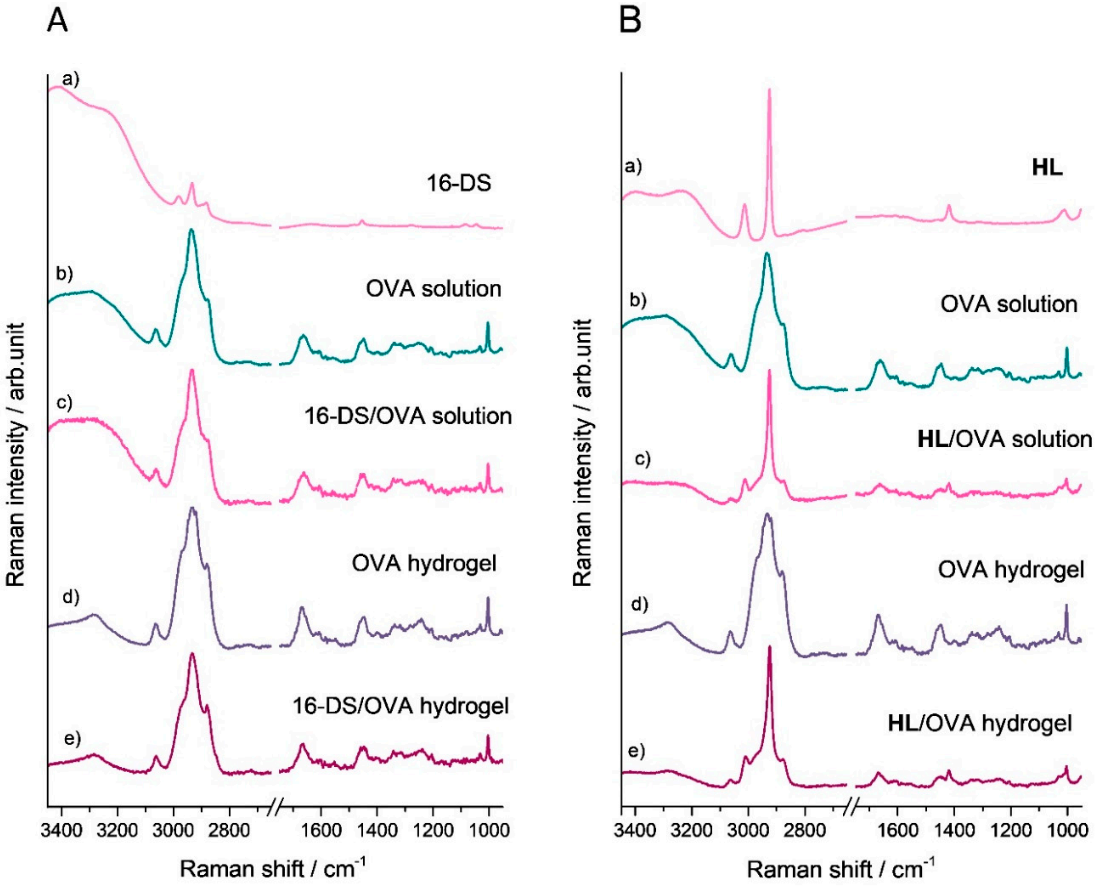

Figure S4. Raman spectra of **A**: (a) 16-DS, (b) 30 *wt*% OVA aqueous solution, (c) 30 *wt*% OVA aqueous solution containing 16-DS in the molar ratio 1:1, (d) 30 *wt*% OVA hydrogel, and (e) 30 *wt*% OVA hydrogel containing 16-DS in the molar ratio 1:1; **B**: (a) **HL**, (b) 30 *wt*% OVA aqueous solution, (c) 30 *wt*% OVA aqueous solution containing **HL** in the molar ratio 10:1, (d) 30 *wt*% OVA hydrogel, and (e) 30 *wt*% OVA hydrogel containing **HL** in the molar ratio 10:1.

5. ATR-FTIR spectra of OVA

The ATR-FTIR spectra of pure OVA samples in the 4000-1200 cm^{-1} region were collected using Nicolet iS20 FTIR spectrometer with a diamond crystal plate, (Thermo Scientific, USA) with 16 scans per spectrum and a 4 cm^{-1} resolution. OMNIC software (Version 9.9.509) was used for advanced ATR correction. Background spectra were collected before each sample spectrum. The amide I and amide II bands, two major bands of the protein IR spectrum, are positioned at 1634 cm^{-1} and 1533 cm^{-1}, respectively. The amide I band is mainly associated with the C=O stretching vibration, and is directly related to the backbone conformation. Amide II results from the N-H bending and the C-N stretching vibrations, and it is not very sensitive to the secondary structure changes. The amide III bands, which are predominantly due to the in-phase combination of N–H in-plane bending and C–N stretching vibrations, are highly sensitive to the secondary structure folding [3], but are ~5–10-fold weaker than those of the amide I and II bands [4]. The amide A (~3288 cm^{-1}) and amide B (~3077 cm^{-1}) bands, which correspond to N-H stretching vibrations, are clearly

visible in the spectrum of the solid form of OVA, but are overlapped by strong vibrations of water in the spectra of OVA solution and hydrogel.

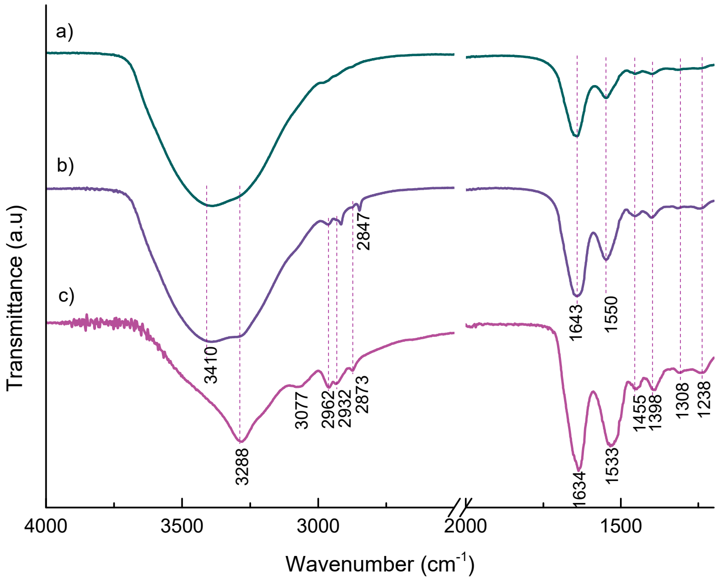

Figure S5. ATR-FTIR spectra of OVA: (**a**) 30 *wt*% aqueous solution, (**b**) 30 *wt*% hydrogel, and (**c**) solid form.

References

1. I.D. Campbell, R.A. Dwek, Biological Spectroscopy, Addison-Wesley, Wokingham, UK (1984).

2. A. Vesković, Đ. Nakarada, A. Popović Bijelić, A novel methodology for hydrogel water content determination by EPR: The basis for real-time monitoring of controlled drug release and hydrogel swelling and degradation, *Polymer Testing*, 98, 107187 (2021).

3. A. Barth, Infrared spectroscopy of proteins, *Biochimica Biophysica Acta*, 1767, 1073-1101 (2007).

4. S. Cai, B.R. Singh, A Distinct Utility of the Amide III Infrared Band for Secondary Structure Estimation of Aqueous Protein Solutions Using Partial Least Squares Methods, *Biochemistry*, 43(9), 2541-2549 (2004).
